# Supplementary material for: Contemplative Practices Behavior Is Positively Associated with Well-Being in Three Global Multi-Regional Stanford WELL for Life Cohorts
Source: Int J Environ Res Public Health. 2022 Oct 18;19(20):13485. doi: 10.3390/ijerph192013485 (PMC9603492; doi:10.3390/ijerph192013485)
Supplement: Supplementary file 1 [file ijerph-19-13485-s001.zip › ijerph-1929776-supplementary.pdf]

Physical Activity Survey Item (CA/Bay Area and New Taipei City): L-Cat 2.2 (Kiernan et al. 2013)

"Think about your physical activity. During the past month, which statement best describes the kinds of physical activity you usually did? Do not include the time you spent working at a job. Please read all six statements before selecting one.

1. I did not do much physical activity. I mostly did things like watching television, reading, playing cards, or playing computer games. Only occasionally, no more than once or twice a month, did I do anything more active such as going for a walk or playing tennis.
2. Once or twice a week, I did light activities such as getting outdoors on the weekends for an easy walk or stroll. Or once or twice a week, I did chores around the house such as sweeping floors or vacuuming.
3. About three times a week, I did moderate activities such as brisk walking, swimming, or riding a bike for about 15-20 minutes each time. Or about once a week, I did moderately difficult chores such as raking or mowing the lawn for about 45-60 minutes. Or about once a week, I played sports such as softball, basketball, or soccer for about 45-60 minutes.
4. Almost daily, that is five or more times a week, I did moderate activities such as brisk walking, swimming, or riding a bike for 30 minutes or more each time. Or about once a week, I did moderately difficult chores or played sports for 2 hours or more.
5. About three times a week, I did vigorous activities such as running or riding hard on a bike for 30 minutes or more each time.
6. Almost daily, that is, five or more times a week, I did vigorous activities such as running or riding hard on a bike for 30 minutes or more each time."

Table S1. Further Characteristics of CA/Bay Area Cohort.

	**N (%)**
**Ethnicity**	
Non-Hispanic	5713 (88.7)
Hispanic	687 (10.7)
Unknown	42 (0.7)
**Race**	
White/Caucasian	3882 (88.7)
Asian or Pacific Islander	1298 (21.7)
Black/African American	205 (3.2)
Multiracial/Multiracial/Another Race	846 (13.1)
Unknown	111 (1.7)
**Region**	
San Francisco Bay Area	4463 (69.3)
Other California	446 (6.9)
Outside California	1420 (22.0)
Unknown	113 (1.8)
**Total**	**6,442 (100.0)**
